# Blood-Brain Barrier Opening in Primary Brain Tumors with Non-invasive MR-Guided Focused Ultrasound: A Clinical Safety and Feasibility Study

**DOI:** 10.1038/s41598-018-36340-0

**Published:** 2019-01-23

**Authors:** Todd Mainprize, Nir Lipsman, Yuexi Huang, Ying Meng, Allison Bethune, Sarah Ironside, Chinthaka Heyn, Ryan Alkins, Maureen Trudeau, Arjun Sahgal, James Perry, Kullervo Hynynen

**Affiliations:** 1 Division of Neurosurgery, Sunnybrook Health Sciences Centre, Toronto, Canada; 2 Hurvitz Brain Sciences Research Program, Sunnybrook Research Institute, Toronto, Canada; 3 Sunnybrook Research Institute, Sunnybrook Health Sciences Centre, Toronto, Canada; 4 Odette Cancer Centre, Sunnybrook Research Institute, Toronto, Canada; 5 Department of Medical Imaging, Sunnybrook Health Sciences Centre, Toronto, Canada; 6 Division of Neurosurgery, Queen’s University, Kingston, Canada; 7 Department of Radiation Oncology, Sunnybrook Health Sciences Centre, Toronto, Canada; 8 Division of Neurology, Sunnybrook Health Sciences Centre, Toronto, Canada; 9 Department of Medical Biophysics, University of Toronto, Toronto, Canada; 10 Institute of Biomaterials and Biomedical Engineering, University of Toronto, Toronto, Canada

## Abstract

The blood-brain barrier (BBB) has long limited therapeutic access to brain tumor and peritumoral tissue. In animals, MR-guided focused ultrasound (MRgFUS) with intravenously injected microbubbles can temporarily and repeatedly disrupt the BBB in a targeted fashion, without open surgery. Our objective is to demonstrate safety and feasibility of MRgFUS BBB opening with systemically administered chemotherapy in patients with glioma in a phase I, single-arm, open-label study. Five patients with previously confirmed or suspected high-grade glioma based on imaging underwent the MRgFUS in conjunction with administration of chemotherapy (n = 1 liposomal doxorubicin, n = 4 temozolomide) one day prior to their scheduled surgical resection. Samples of “sonicated” and “unsonicated” tissue were measured for the chemotherapy by liquid-chromatography-mass spectrometry. Complete follow-up was three months. The procedure was well-tolerated, with no adverse clinical or radiologic events related to the procedure. The BBB within the target volume showed radiographic evidence of opening with an immediate 15–50% increased contrast enhancement on T1-weighted MRI, and resolution approximately 20 hours after. Biochemical analysis of sonicated versus unsonicated tissue suggest chemotherapy delivery is feasible. In this study, we demonstrated transient BBB opening in tumor and peritumor tissue using non-invasive low-intensity MRgFUS with systemically administered chemotherapy was safe and feasible. The characterization of therapeutic delivery and clinical response to this treatment paradigm requires further investigation.

## Introduction

Global efforts to improve the prognosis for patients with glioblastoma (GBM) have been met with limited success. The median survival time remains at approximately 15 months following surgical resection and Temozolomide (TMZ) chemotherapy concurrent with radiotherapy^1^. The lethality of brain tumors remains high relative to other cancers, in part because penetration of the central nervous system (CNS) by systemic agents is restricted by the blood-brain barrier (BBB). While the BBB is dysfunctional in many malignant brain tumors, its integrity has been shown to be variable by dynamic contrast enhanced MRI. Further out in the peritumor tissue, the BBB remains intact but invasive tumor cells are present and remain after surgical resection. Chemotherapy concentrations, such as carboplatin and paclitaxel, within the peritumor tissue are up to 40 times lower than at the tumor centre^2–4^.

Various methods to overcome the BBB have been investigated though each with disadvantages that preclude successful translation to patients. Direct intracranial injection or convection-enhanced delivery can improve drug concentrations at the target, but also have safety concerns of open surgery^5^. Modification of therapeutics to bypass the BBB via human insulin receptors has been shown to have low spatial specificity and off-target effects posing safety concerns in non-human primate studies^6^. Minimally invasive surgery is attractive to patients for improved recovery time and certain surgical risks such as hemorrhage and infection. Stereotactic radiation and MR-guided focused ultrasound (MRgFUS) are two minimally invasive methods of disrupting the BBB with high spatial resolution. Although increased BBB permeability is achievable with a small dose of radiation, the time frame to maximal disruption is unknown and recovery may take as long as 90 days^7^.

In transcranial non-invasive MRgFUS, ultrasound from 1024 individually driven transducer elements surrounding the skull under real-time image guidance, is delivered with sub-millimeter accuracy. While thermoablation using heat generated by high-intensity ultrasound appears to be the most straightforward approach to treating brain tumors, difficulties lie in achieving adequate tumor necrosis and minimizing off-target effects that might result in tissue damage or hemorrhage^8,9^. Low-intensity ultrasound, delivers <0.1% of the energy required for thermoablation by interacting with intravenously injected microbubbles to create a temporary disruption of the BBB^10^. Due to the lower energy requirement, the volume of BBB disruption can be expanded, and customized for shape and location within the intracranial vault.

In animal studies, BBB opening has been shown to be immediate, repeatable, resolve within six to eight hours, and not cause axonal or neuronal injury^11^. Furthermore, enhanced delivery of trastuzumab^12^, doxorubicin^13^, TMZ^14^, methotrexate^15^, as well as viruses^16^ and cells^17^ has been demonstrated in small to large animal models. Animal studies looking at clinically relevant outcomes show longer median survival of rats with 9 L gliomas after three weekly treatments of FUS aided doxorubicin^18^, as well as longer survival of rats with HER-2 amplified brain tumors after FUS delivered NK-92 cells with HER2 specific receptors^19^. Furthermore, P-glycoprotein expression, a common multi-drug resistant protein in the BBB responsible for efflux of various chemotherapeutic agents, is decreased after BBB disruption^20^. Patients with brain tumors may significantly benefit from a modality capable of precise targeting of BBB disruption.

A surgically implanted pulsed ultrasound system has recently been used for BBB disruption in conjunction with systemic microbubbles. Carboplatin delivery through this method was well tolerated in patients with recurrent GBM^21^, and was not associated with clinical or radiographic adverse events. MRgFUS is different in that it does not require open surgery and provides fine spatial control over the treatment field and uniformity of the BBB opening. Our primary objective is to determine the safety and feasibility of opening the BBB in peritumor brain tissue using transcranial low-intensity MRgFUS during the administration of systemic chemotherapy, with a secondary aim to quantify drug levels in sonicated and unsonicated tissue. This is the first report of targeted chemotherapy delivery using MRgFUS and has significant implications for future neuro-oncology and surgical trials and practice.

## Results

**Figure 1:**
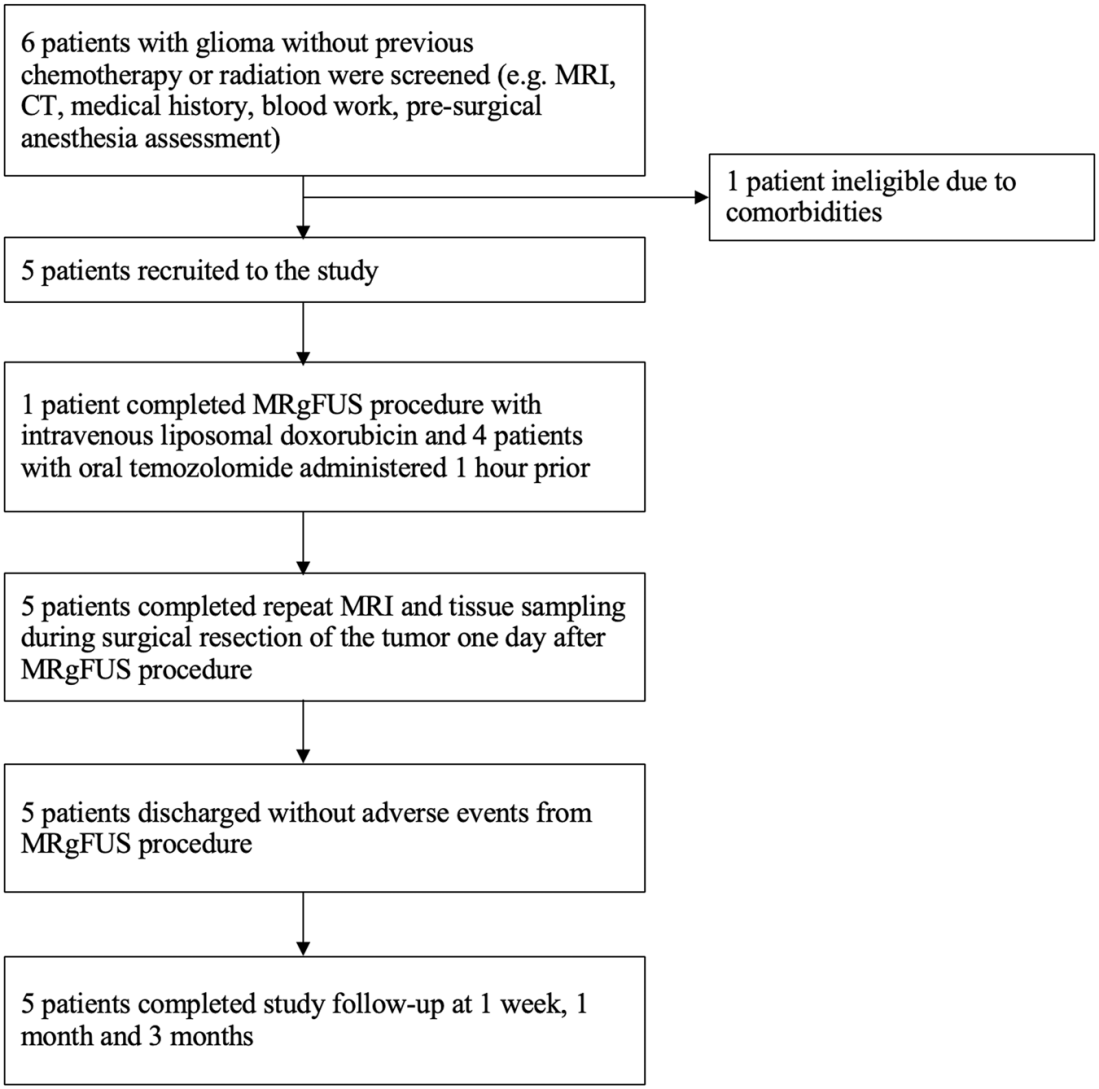
Overview of study.

Between 2015 and 2017, five patients with malignant gliomas were enrolled in this study (Fig. 1). Participant demographics are outlined in Table 1. Patient five had a previous craniotomy, while none of the other patients had previous intracranial surgeries. Throughout the three-month follow-up, all patients received standard neuro-oncology care. Detailed treatment parameters are listed in Table S1.

**Table 1:** Patient Demographics. Pathology as confirmed by post-surgical pathology report.

Patient	Age	Sex	Location	Pathology	Presenting symptoms	Chemotherapy Delivered
1	56	F	Right temporal	Grade III anaplastic astrocytoma	Seizures	IV liposomal Doxorubicin, 58 mg
2	62	M	Right parieto-occipital	Grade IV astrocytoma	Headache, visual changes	PO Temozolomide, 160 mg
3	71	M	Right temporal	Grade IV astrocytoma	Leg weakness, headache	PO Temozolomide, 140 mg
4	57	M	Right frontal	Grade IV astrocytoma	Seizures	PO Temozolomide, 140 mg
5	33	M	Right frontal	Grade III anaplastic astrocytoma	Previous craniotomy 2016, Seizures, recurrent tumor	PO Temozolomide, 140 mg

All patients underwent MRgFUS BBB disruption procedure with no clinically significant ultrasound related clinical or radiologic adverse events (e.g. intracerebral haemorrhage or edema). Specifically, the sonication procedure itself was well tolerated with no new or worsening symptoms in the 24 hours between MRgFUS procedure and tumor resection. One patient aborted prior to the final sonication target due to back pain on the MRI table. Minor headache at the helmet attachment sites was reported in two patients; this resolved in both prior to the surgical procedure the following day. Following resection of right temporal tumor, patient one suffered a post-operative left superior quadrant hemianopsia from the sacrifice of a temporal artery traversing the tumor. The neurological exam post FUS and preceding surgery were normal. The surgically induced deficit resolved, and visual fields were full to confrontation at post-operative day 14.

**Figure 2:**
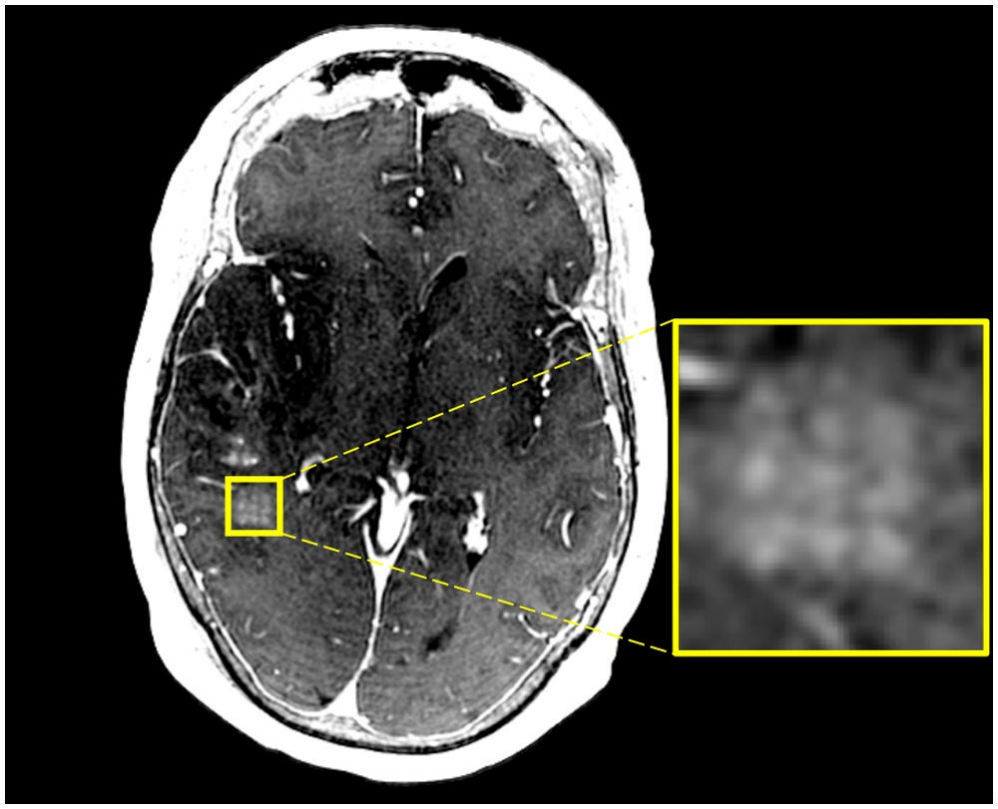
Axial T1-weighted post gadolinium MRI of patient one immediately after MRgFUS BBB disruption demonstrates contrast extravasation in the grid pattern (see enlargement) where sonication occurred. The contrast extravasation is discrete and precise. There is no evidence of edema secondary to the procedure.

**Figure 3:**
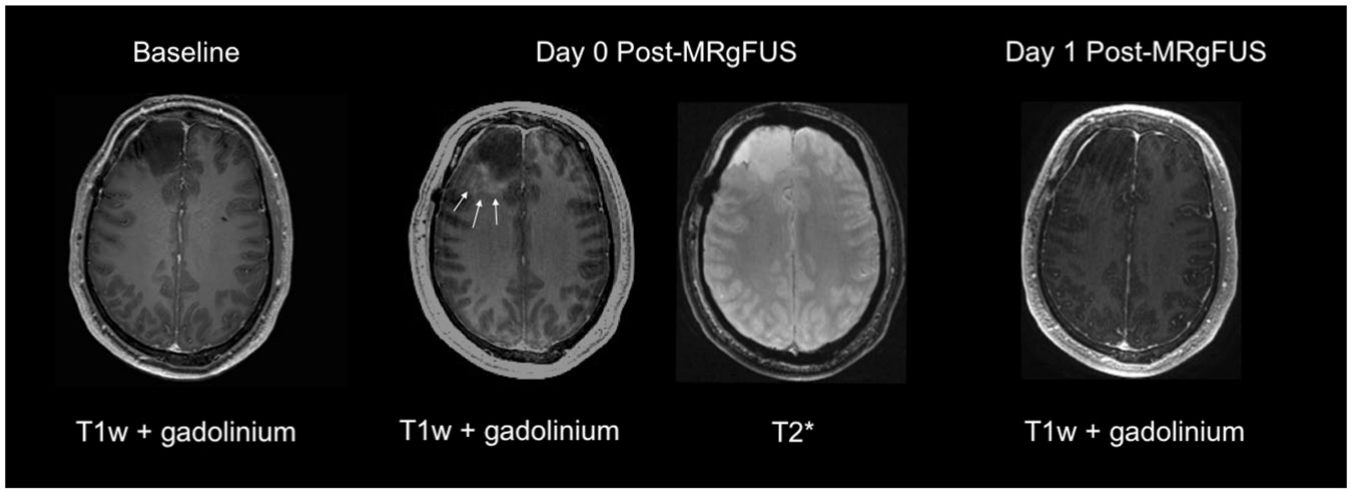
Sample T1 weighted post-gadolinium MRI from patient five obtained 30 days prior to BBB opening procedure (left), immediately following BBB disruption (middle) and 20 hours post BBB disruption (right). Ill-defined contrast enhancement is seen in the peritumoral region on images acquired immediately after MRgFUS (white arrows). This contrast enhancement has resolved in the peritumor region on the day 1 follow-up image indicating closure of the BBB. T2* sequence acquired immediately following BBB disruption for this patient show no evidence of microhemorrhages.

The BBB was safely and successfully opened in the five patients enrolled, as shown by post-sonication gadolinium enhancement in the target region and resolution twenty-fours after (Figs 2 and 3). Table 2 indicates percent change in signal intensity in region of interest (sonicated) relative to adjacent non-enhancing tumor margin tissue in the ipsilateral hemisphere. BBB opening was achieved in a range of two to five standard sonication volumes (486 mm^3^) per patient. In patient two, contrast enhancement was not identifiable, however, we observed cavitation signals indicating BBB opening from intra-procedural acoustic feedback. BBB opening was achieved predictably using 50% of the power at which cavitation was observed during a ramp test in the final two participants.

**Table 2:** Summary of contrast enhancement and drug delivery after blood-brain barrier disruption.

Patient	Sonicated volume (mm^3^)	Sonication power (W)	% Enhancement	Chemotherapy concentration in peritumor tissue	
				sonicated (ng/mg)	unsonicated (ng/mg)
1	486 × 2	5–9	40 ± 11	0.22	0.15
2	486 × 4	6–7.5	—	—	—
3	486 × 5	8–10	50 ± 22	—	—
4	486 × 2	6–7.5	15 ± 5	3.47 × 10^−4^	0.45 × 10^−4^
5	486 × 5	4–15	35 ± 15	—	—

Percent enhancement is the signal intensity in sonicated tissue versus non-enhancing adjacent tissue of the ipsilateral hemisphere. The range of power is provided for the bouts of sonication delivered for each individual patient. Note patient one received intravenous liposomal doxorubicin, and patient two to five received oral temozolomide.

Peritumor chemotherapy levels were quantifiable in patients one and four. Of note, patient one received intravenous liposomal doxorubicin while patient four oral TMZ. The volume of resectable tissue sample for patients two, three, and five was limited, which prevented quantitative analysis of tumor margin samples. A trend of elevated concentration in sonicated tissue relative to unsonicated tissue is observed in the peritumor regions of these participants (Table 2).

## Discussion

Our study is a demonstration of safety in BBB opening in patients with malignant brain tumor using noninvasive, transcranial MRgFUS. This sample suggests controlled image-guided BBB disruption appears both safe, and reversible, and well tolerated among this preliminary group of patients. Although an implanted pulsed ultrasound transducer has been recently studied in patients with malignant brain tumors^21^, our technology employs a transcranial device and is therefore incisionless. MRgFUS can be applied through an intact skull, thereby eliminating an invasive procedure and cortical bone implants. Furthermore, tailoring of the precise target location and size is possible with MRgFUS, while an implanted device will limit the BBB opening to direction of the transducer. Furthermore, neuro-oncology patients often will have had previous craniotomies. For these patients, such as patient five, MRgFUS provides an additional patient tailoring, through selective deactivation of transducers at sites of cranial bone fixation.

Chemotherapy concentration in sonicated and unsonicated peritumor tissue was assessed for feasibility of improved delivery by MRgFUS BBB disruption. Due to anatomic constraints and surgical exposure, tissue sampling was unfortunately unavailable or minimal in three of these patients. In the two patients with measurable peritumor results, chemotherapy concentration was higher in the tissue where BBB disruption occurred in contrast to unsonicated (non-BBB disrupted) tissue, with the caveat that TMZ concentration found in patient four fell outside the range of detection. The different ranges in concentration between the patients’ measured may be explained by the change in chemotherapy and route of administration. Finally, given the half-life of TMZ is approximately 1.8 hours, the measurable volume of delivery is likely underrepresented given the considerable time between drug administration and surgical sampling.

We acknowledge several limitations of this study, which are the small size, change in chemotherapy from liposomal doxorubicin to TMZ, and missing data preventing conclusions about the exploratory variables of chemotherapy concentration. Precision errors in sampling of the relatively small sonicated tissue volume during a craniotomy inherently complicate such interpretations. Nevertheless, the primary objective of safety determination in this first-in-human proof-of-concept study was achieved. Safe, temporary BBB disruption in tumor and peritumor tissue using a targeted, non-invasive method was demonstrated. Furthermore, procedural knowledge was advanced by this study, in the establishment of a ramp protocol for determining the optimal sonication power, and is described in further detail in Huang *et al*.^22^ and O’Reilly and Hynynen^10^. Our experience and results from this trial support the generalizability of MRgFUS in neuro-oncological applications. Improving access of chemotherapy to peritumor tissue may be incorporated, and in fact should be a critical consideration, in adjuvant therapies. Specifically, further trials will aim to i) reproduce these results in greater number of patients, ii) deliver a range of therapeutic agents to a variety of presently inaccessible brain tumors, and iii) modify sonication parameters to tailor BBB disruption to various brain tissues, tumor and otherwise.

## Methods

### Study design and participants

This study was a prospective single-arm, open-label design with the aim of evaluating safety and feasibility of opening the BBB in patients with brain tumor using MRgFUS. The secondary aim of this study was to evaluate the feasibility of chemotherapy delivery using low-intensity MRgFUS. All patients screened were identified through an outpatient neurosurgery clinic, neuro-oncology referral, or the emergency department. Participants consented for surgical resection prior to undergoing a separate discussion of informed consent for research participation. This study and all its methods were approved by and conducted in accordance with the Research Ethics Board at Sunnybrook Health Sciences Centre and Health Canada. The study was registered on 22/01/2015 with identifier NCT02343991.

The study flow is outlined in Fig. 1. Eligible patients between 18 and 80 years with radiographic evidence of malignant glioma and Karnofsky Performance Status score of 70–100 were included. All patients had consented for surgical resection prior to discussing study participation. Patients with previous irradiation or full course of chemotherapy, evidence of significant mass effect or increased intracranial pressure were excluded. Full exclusion criteria are outlined in Table 3. Key exclusions were contraindication to MRI or ultrasound contrast Definity® (e.g. significant uncontrolled pulmonary disease), significant cardiac or renal diseases, and abnormal coagulation factors increasing the risk intracranial hemorrhage. All participants were screened by the anesthesia team and underwent MRI and CT scans for intra-procedural image registration and target planning.

**Table 3:** Key inclusion and exclusion criteria.

Inclusion	Exclusion
Men or women 18 to 75 years, inclusive Able and willing to give informed consent Malignant brain tumor confirmed by biopsy or suspected based on imaging Tumor is clearly defined on pre-therapy contrast enhanced MRI scans Size of the targeted portion of the tumor (i.e. prescribed ROT) is less than 2.5 cm in diameter (16 cm^3^). The non-targeted tumor tissue may exceed the targeted volume Karnofsky rating 70-100 ASA score 1–3 At least 14 days passed since last brain surgery	Previously irradiated tumor or tumor site Previous full course of chemotherapy Contraindication to MRI Tumor presenting with the following imaging characteristics: brain edema or mass effect with midline shift > 10 mm after steroid treatment, recent intracranial hemorrhage Evidence of increased intracranial pressure Unstable cardiovascular or pulmonary disease, cardiac shunt Abnormal coagulation Cerebral or systemic vasculopathy Insulin-dependent diabetes mellitus

### MR-guided focused ultrasound procedure

The ExAblate Neuro (InSightec Tirat Carmel, Israel) system was used for transcranial focused ultrasound delivery. The stereotactic frame attached to the ultrasound helmet containing 1024 transducers at the centre frequency of 220 kHz, with coupling to a 3T MR scanner (Signa MR750, GE Healthcare, Milwaukee, WI, USA). The device enabled intraoperative imaging and real-time acoustic feedback determining sonication parameters.

Following a full head shave, the participant’s head was fitted with a stereotactic frame. One hour prior to sonication, participants were systemically administered a sub-therapeutic dose of chemotherapy. The administration was timed so the concentration would be maximal concentration at the expected time of BBB opening. The participant was placed supine on the MRI table, kept awake and given an emergency switch to abort the procedure in case of discomfort or pain. Anaesthesia services were available on standby to provide mild sedation or pain medication as needed.

Pre-sonication T1, T2 (Fast Spin Echo) and T2* MR sequences were acquired for baseline and target planning. The sonication volumes were delineated by a cubic 3-by-3 grid with 3 mm spacing, totalling approximately 9 × 9 × 6 mm^3^. Fig. S1 depicts production of the grid. Sonication volumes were placed at tumor margins aligned with the surgical trajectory for tumor resection the following day. Once the target regions were identified, the participant received an intravenous injection of Definity® (4 μl/kg) immediately preceding sonication at each target location. The total dose of Definity® did not exceed 20 μl/kg. Optimal power for BBB opening was calculated as 50% of the power at which cavitation signals were first detected using acoustic feedback from an incremental sonication power protocol. Each sonication was delivered at 0.74% duty cycle for 50 seconds, and further details are described by Huang *et al*.^22^.

Following the completion of sonications, a gadolinium enhanced T1-weighted MRI was performed to confirm BBB opening. Contrast enhancement at the targeted regions signified the end of the procedure. Participants were admitted to a neurosurgical ward for observation and for subsequent tumor resection the next day. A follow-up MRI with gadolinium was performed the morning after MRgFUS procedure prior to open surgery to ensure BBB closure. All patients underwent the planned craniotomy for tumor resection in the standard fashion.

### Outcomes

The primary outcomes were safety as assessed by clinical neurologic exam and radiologic evidence of haemorrhage, swelling or mass effect, as well as technical feasibility determined by contrast enhancement in the target regions with resolution within the following twenty-four hours. Follow up visits were scheduled for one day, one week, and one month and three months after the MRgFUS procedure (Fig. 1). Contrast enhancement in the sonicated volume was quantified on MRI by the percentage change in the T1-weighted signal intensity within the defined region of interest (ROI) compared to an ROI of the same volume in the adjacent unsonicated tissue. The ROIs were manually delineated, and signal intensity extracted using OsiriX MD Lite.

A preliminary assessment of drug delivery feasibility using this technique was assessed by measuring the concentration of chemotherapy in tissue samples taken during surgery. Surgical resection of the tumor and sonicated and unsonicated peritumor tissues took place approximately 24 hours after chemotherapy administration and FUS. Prior to tumor resection, needle biopsy of the sonicated and unsonicated regions was performed using a frameless stereotaxic system. The targets were defined on the post sonication T1 with contrast enhanced MRI. The patients then underwent standard craniotomies and maximal safe tumor resection. Samples were stored at −80 °C. It should be noted that following the first participant, the protocol for chemotherapy agent was amended to more closely align the research methods with the clinical care. Participant 1 received intravenous liposomal doxorubicin, and the remaining received TMZ.

### Biochemical analysis

Chemotherapy concentrations were quantified using liquid chromatography-mass spectrometry performed by the Analytical Facility for Bioactive Molecules, The Hospital for Sick Children, Toronto, Canada. Briefly, standards in 0.1 N HCl were spiked into control pig brain homogenized in 0.1 N N HCl at 10 mg/100uL. Internal standard (Temozolomide-d4, Toronto Research Chemicals) 100 ng/ml, was spiked into standards and samples (100 uL brain homogenate −10mg/100uL in 0.1 N N HCl). 2 mL ethyl acetate (Caledon) was then added. Samples and standards were then vortexed for one minute and centrifuged at 800 x G for 10 minutes. Supernatant was removed and transferred to a conical tube and then taken to dryness under a gentle stream of Nitrogen. Samples and standards were reconstituted in 1 mL 10/90 water/acetonitrile 5 mM ammonium formate pH 3.2. Extracted samples and standards were analyzed on a Sciex QTrap 5500 with an Agilent 1290 HPLC using a Phenomenex Kinetex HILIC 2.6 µm 100 Å 50 × 4.6 mm column. Samples were eluted using a gradient flow of A) 90/10 water/acetonitrile 5 mM ammonium formate pH 3.2 and B) 10/90 water/acetonitrile 5 mM ammonium formate pH 3.2 over a period of 5 minutes as follows: t = 0 min −100%B, t = 2 min −100%B, t = 3 min 50%B, t = 3.5 min – 100%B t = 5 min 100%B. Data was collected and analyzed using Sciex Analyst v 1.6.3. The range of detection for doxorubicin is 0.1–100 ng/mg, and for temozolomide 0.001–5 ng/mg.

## Electronic supplementary material

Supplementary material

## Data Availability

Data can be made available upon reasonable request to corresponding author Dr. Todd Mainprize.
