# Supplementary material for: Blood-Brain Barrier Opening in Primary Brain Tumors with Non-invasive MR-Guided Focused Ultrasound: A Clinical Safety and Feasibility Study
Source: Sci Rep. 2019 Jan 23;9:321. doi: 10.1038/s41598-018-36340-0 (PMC6344541; doi:10.1038/s41598-018-36340-0)
Supplement: Supplementary file 1 — Supplementary material [file 41598_2018_36340_MOESM1_ESM.pdf]

Supplementary Information

Blood-Brain Barrier Opening in Primary Brain Tumors with Non-invasive MR-Guided Focused Ultrasound: A Clinical Safety and Feasibility Study

Todd Mainprize, Nir Lipsman, Yuexi Huang, Ying Meng, Allison Bethune, Sarah Ironside, Chinthaka Heyn, Ryan Alkins, Maureen Trudeau, Arjun Sahgal, James Perry, Kullervo Hynynen

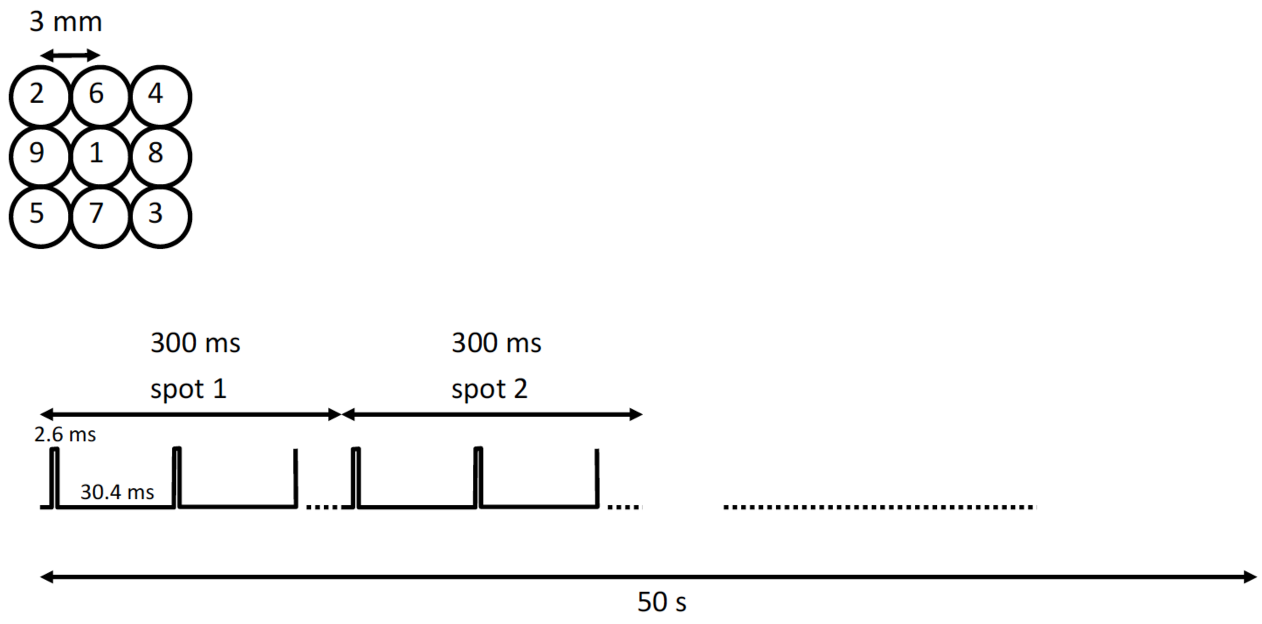

Figure S1.

Sonication grid: spatial and temporal representation of each of nine targeted ultrasound spots within a sonication grid.

Patient	No. of Targets	Grid Dimensions [a] (cm^3)	Range of Actual Sonication Power (Watts)	Maximal Sonication Duration (Seconds)
1	2	0.9 x 0.9 x 0.6	5-9	50 s repeated
2	4	0.9 x 0.9 x 0.6	6-7.5	50 s repeated
3	5	0.9 x 0.9 x 0.6	8-10	50 s repeated
4[b]	2	0.9 x 0.9 x 0.6	6-7.5	50 s repeated
5	5	0.9 x 0.9 x 0.6	4-15	50 s repeated

Table S1.

Sonication parameters

[a] Grid dimensions: (Anterior-Posterior x Medial-Lateral x Superior-Inferior).

[b] Patient aborted prior to central tumor sonication due to back pain
